# Adherence to the Mediterranean Diet and Risk of Gastric Cancer: A Systematic Review and Meta-Analysis

**DOI:** 10.3390/nu15173826

**Published:** 2023-09-01

**Authors:** Xiao Bai, Xue Li, Siqi Ding, Dongqiu Dai

**Affiliations:** 1 Department of Surgical Oncology, The Fourth Affiliated Hospital of China Medical University, Shenyang 110032, China; xiaobai@cmu.edu.cn (X.B.); 2021121681@cmu.edu.cn (X.L.); 2021121602@cmu.edu.cn (S.D.); 2 Cancer Center, The Fourth Affiliated Hospital of China Medical University, Shenyang 110032, China

**Keywords:** Mediterranean diet, gastric cancer, meta-analysis

## Abstract

Available results on the association between the Mediterranean diet (MD) and gastric cancer (GC) incidence are controversial. The present study aimed to determine the correlation between different subtypes of GC and MD adherence. This meta-analysis was registered on PROSPERO (CRD42021284432). We searched Embase, PubMed, Cochrane Library, and Web of Science from inception through 22 April 2023 to retrieve relevant studies. A random-effects model was used to pool odds ratios (ORs) with 95% confidence intervals (CIs). Eleven studies were included in the meta-analysis. Pooled analyses revealed that adherence to the MD was inversely associated with GC risk (OR_cc_, 0.43; 95% CI, 0.29 to 0.63; OR_coh_, 0.84; 95% CI, 0.77 to 0.92). Higher MD adherence was significantly associated with a reduced GC risk in male (OR_cc_, 0.78; 95% CI, 0.65 to 0.93; OR_coh_, 0.81; 95% CI, 0.65 to 1.01), but not in female (OR_cc_, 0.83; 95% CI, 0.68 to 1.01; OR_coh_, 1.04; 95% CI, 0.82 to 1.31). Furthermore, adherence to the MD possibly decreased the risk of gastric cardia adenocarcinoma (GCA) (OR_cc_, 0.64; 95% CI, 0.49 to 0.83; OR_coh_, 0.88; 95% CI, 0.76 to 1.02) and gastric non-cardia adenocarcinoma (GNCA) (OR_cc_, 0.68; 95% CI, 0.59 to 0.79; OR_coh_, 0.85; 95% CI, 0.78 to 0.94). Our results indicate that adherence to the MD reduces the risk of GC and its subtypes.

## 1. Introduction

Gastric cancer (GC) is a serious health concern worldwide. According to the International Agency for Research on Cancer’s data on the latest global burden of cancer, new GC cases in the world reached over one million in 2020, ranking fifth among the overall new cancer cases [1]. GC is a complex cancer that develops due to multiple factors. Although family history is an important risk factor, studies have shown that some modifiable risk factors, including diet, alcohol, and smoking, may have a greater influence on the development of GC in countries with an increasing prevalence of GC [2]. It may be possible to reduce the risk of GC by adopting appropriate diets and changing to healthy lifestyles.

Diet and multiple chronic non-communicable diseases are strongly linked [3,4]. Increasing studies have explored the relationship between dietary patterns and GC risk, including the most representative Mediterranean diet (MD) pattern. The MD pattern is characterized by eating more vegetables, grains, fruits, legumes, nuts, olive oil, and fish; consuming fewer dairy products and meat; and drinking moderate amounts of alcohol [5]. Some studies showed that the components of MD can prevent GC by inhibiting tumor angiogenesis and reducing DNA damage [6,7]. However, other studies had contradictory hypotheses about the role of MD in GC development [8,9]. Therefore, the correlation between MD adherence and GC risk is still unclear, and it is essential to explore it further using a systematic review and meta-analysis approach.

Furthermore, GC can be categorized into gastric non-cardia adenocarcinoma (GNCA) and gastric cardia adenocarcinoma (GCA) according to the anatomical site [10]. The two subtypes of GC differ in both pathogenetic mechanisms and their risk factors [11]. To date, there is no meta-analysis to comprehensively clarify the effect of high MD adherence on GC risk and the correlation between different subtypes of GC and MD adherence. We therefore performed this study to understand whether high MD adherence can prevent GC and its subtypes.

## 2. Materials and Methods

### 2.1. Protocol and Registration

This meta-analysis complied with the Preferred Reporting Items of Systematic Reviews and Meta-Analyses (PRISMA) guidelines [12]. It was registered on the PROSPERO International prospective register of systematic reviews (CRD42021284432), which provides more details.

### 2.2. Search Strategy and Selection Criteria

We retrieved all relevant articles published before 22 April 2023, from Embase, Cochrane Library, PubMed, and Web of Science. The specific search strategy is shown in Table S1. The references in the initially identified literature were retrieved manually for other potentially eligible articles.

The following studies were eligible for our study: (1) cohort or case–control studies; (2) studies reporting the association between the MD and the risk of GC; (3) studies on any type of GC; and (4) studies with adjustment of hazard ratios (HRs), relative risks (RRs), or odds ratios (ORs) with corresponding 95% confidence intervals (CIs). If several publications reported similar clinical findings, the one with the most complete data was included in the present analysis.

The exclusion criteria were as follows: (1) non-English publications; (2) unavailability of the full text; and (3) review articles, conference abstracts, letters, case reports, systematic reviews, and meta-analyses.

### 2.3. Data Extraction and Quality Assessment

Two researchers (X.B. and X.L.) independently checked the titles and abstracts of the searched studies and excluded apparently irrelevant articles. Any disagreement was settled by discussion. The following data were extracted from the included studies: author’s name, study design, year of publication, country, population, follow-up (cohort studies), number of cases and controls (case–control studies), age, gender, components of score, adjustment and risk estimates, and supplementary information. The risk estimates mostly included RRs, HRs, and ORs (highest vs. lowest category) with 95% CIs.

In this meta-analysis, two researchers (X.B. and X.L.) used the Newcastle–Ottawa Scale [13] to assess the quality of the included studies. Any disagreement was settled by discussion for reaching a consensus. The quality assessment involved three domains: selection of the research object, comparability, and outcome. Studies were rated from 0 to 9, with the score of 7–9 regarded as high quality.

### 2.4. Statistical Analysis

Stata 16.0 (Stata Corporation, College Station, TX, USA) was used for statistical analysis. A DerSimonian and Laird random-effects model was used to pool multivariate-adjusted ORs with 95% CIs [14,15]. In this study, estimates from cohort and case–control studies were compared separately. OR values of case–control studies (OR_cc_) and cohort studies (OR_coh_) were also represented, respectively. And the estimates of observational studies were derived from the according estimates of both case–control and cohort studies. As is known, cohort studies often have a follow-up time compared to case–control studies, and previous studies have shown a lower effect estimates with increasing follow-up time [16,17]. Additional linear regression analysis was conducted to assess the effect of follow-up time using R version 4.3.1 (R Foundation for Statistical Computing, Vienna, Austria).

Heterogeneity was measured using the chi-squared test (Cochran’s Q test) and I^2^ statistics, with I^2^ values of 25%, 50%, and 75% graded as low, medium, and high heterogeneity, respectively [18]. To further address heterogeneity, we conducted a subgroup analysis [19]. The following two aspects were considered: (1) geographic location and (2) gender. In terms of sensitivity analysis, a single study was excluded each time, and the pooled risk estimates were recalculated. For comparison of studies ≥ 10, publication bias was statistically assessed using Begg’s test [20] and Egger’s test [21]. A two-sided *p* < 0.05 indicated statistically significant differences.

## 3. Results

### 3.1. Literature Search and Study Characteristics

**Figure 1:**
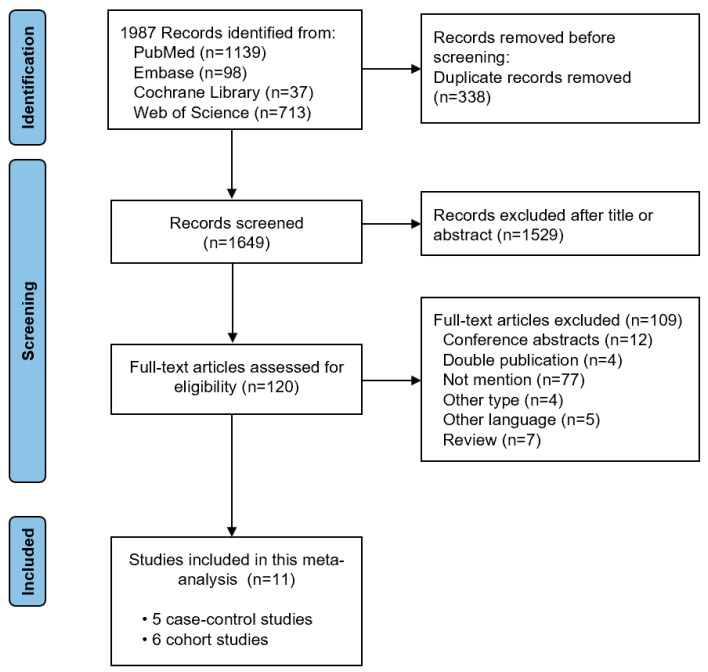
Literature search flow diagram.

During the initial search, 1987 relevant studies were identified. After removing duplicates, the titles and abstracts were checked. Next, 120 full-text articles were evaluated for eligibility. We finally included eleven studies (with five case–control studies and six cohort studies) in this meta-analysis after reviewing the full text [7,8,9,22,23,24,25,26,27,28,29]. The study selection process is shown in Figure 1. The basic characteristics of the included studies and the quality assessment of the included studies is shown in Table 1. The Newcastle–Ottawa Scale score of three case–control studies [25,26,29] and five cohort studies [8,22,23,27,28] was greater than 7, representing high quality research.

### 3.2. Effect of the MD on GC Risk

#### 3.2.1. Estimates from Different Types of Studies

**Figure 2:**
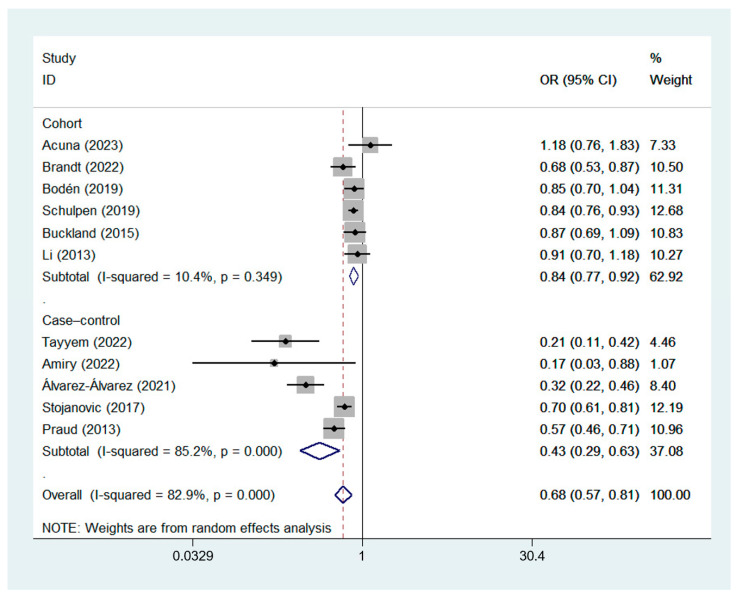
Forest plot of the association between the MD and GC risk in cohort [8,9,22,23,27,28] and case–control studies [7,24,25,26,29].

A random-effects model was used for data analysis, and the results showed that high MD adherence was significantly correlated with a reduced risk of GC (OR_cc_, 0.43; 95% CI, 0.29 to 0.63, I^2^ = 85.2%; OR_coh_, 0.84; 95% CI, 0.77 to 0.92, I^2^ = 10.4%; OR_observational_, 0.68; 95% CI, 0.57 to 0.81, I^2^ = 82.9%) [7,8,9,22,23,24,25,26,27,28,29]. The forest plot of the association between the MD and GC risk is shown in Figure 2.

#### 3.2.2. Effect of Follow-Up Time

**Figure 3:**
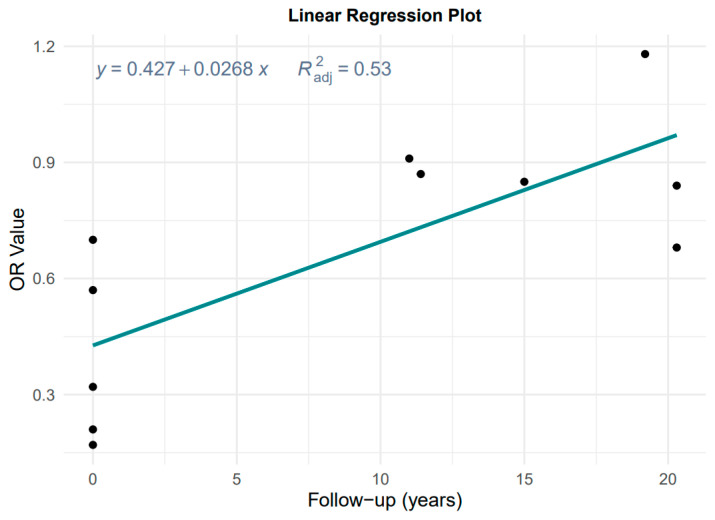
Plot of the linear regression of OR value against follow-up time.

Data from Figure 2 and Table 1 were used to evaluate the effect of follow-up time, considering the degradation of MD effect with a longer follow-up time in cohort studies. Figure 3 shows the result of the plot of OR versus follow-up time.

The linear regression is OR = 0.427 + 0.028 × years, adjusted *R*^2^ = 0.53, *p* = 0.001, which is highly significant. Therefore, it is not reasonable to pool case–control and cohort studies without considering the effect of follow-up time on the effect estimates, and instead we chose to take the results of the according case–control studies as the primary results for MD and the risk of GC and its subtypes.

### 3.3. Effect of the MD on GCA and GNCA Risk

**Figure 4:**
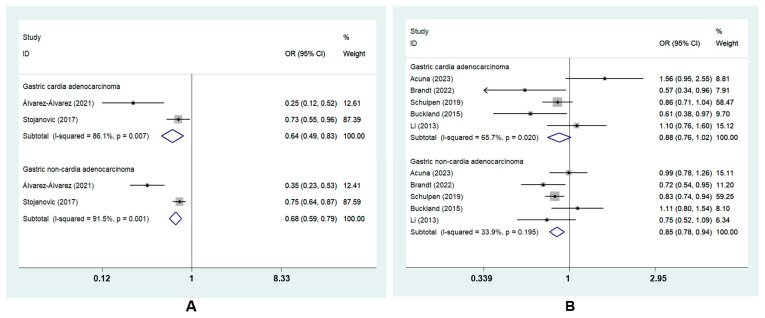
Forest plot of the association between the MD and the risk of GC subtypes in (**A**) case–control studies [7,26] and (**B**) cohort studies [8,22,23,27,28].

GC was divided into two subtypes based on tumor sites: GCA and GNCA. The subgroup analysis revealed that the highest MD adherence was correlated with a lower risk of GCA (OR_cc_, 0.64; 95% CI, 0.49 to 0.83, I^2^ = 86.1%) and GNCA (OR_cc_, 0.68; 95% CI, 0.59 to 0.79, I^2^ = 91.5%; OR_coh_, 0.85; 95% CI, 0.78 to 0.94, I^2^ = 33.9%). However, there was no risk reduction for GCA in cohort studies (OR_coh_, 0.88, 95% CI, 0.76 to 1.02, I^2^ = 65.7%) [8,22,23,27,28] (Figure 4).

### 3.4. Subgroup Analysis of MD and GC Risk

**Figure 5:**
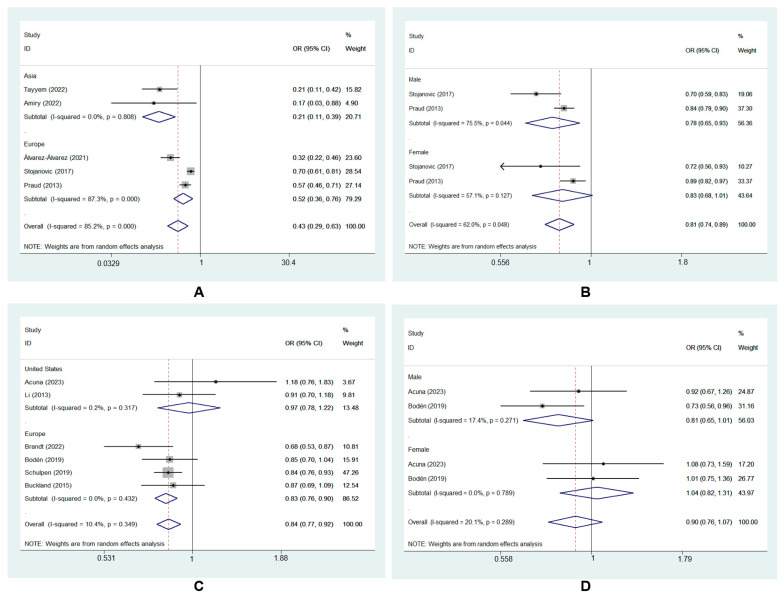
Subgroup analysis of MD and GC risk in case–control studies: (**A**) geographic location [7,24,25,26,29]; (**B**) gender [7,29] and cohort studies: (**C**) geographic location [8,9,22,23,27,28]; (**D**) gender [9,22].

Subgroup analysis was performed to further explore the source of heterogeneity. Neither geographic location nor gender changed the direction of effect estimates, and the results for the source of heterogeneity are presented in Table 2, which mainly showed MD and GC risk in case–control studies here. The results of subgroup analysis for MD and GC risk in cohort studies are shown in Figure 5C,D.

Based on the geographic location of the included studies, the subgroup analysis showed that adherence to the MD significantly reduced GC risk in Asia (OR_cc_, 0.21; 95% CI, 0.11 to 0.39, I^2^ = 0.0%) [24,25]. This protective effect was also found in Europe (OR_cc_, 0.52; 95% CI, 0.36 to 0.76, I^2^ = 87.3%) [7,26,29] (Figure 5A).

The subgroup analysis also showed that the MD had a protective effect on GC in males (OR_cc_, 0.78; 95% CI, 0.65 to 0.93, I^2^ = 75.5%) [7,29]. However, no significant effect was found in females (OR_cc_, 0.83; 95% CI, 0.68 to 1.01, I^2^ = 57.1%) [7,29] (Figure 5B).

Subgroup analyses showed that the Asia and female subgroups had lower heterogeneity (I^2^ = 0.0% and 57.1%, respectively). This finding indicated that geographic location and gender does explain the source of heterogeneity.

### 3.5. Sensitivity Analysis

**Figure 6:**
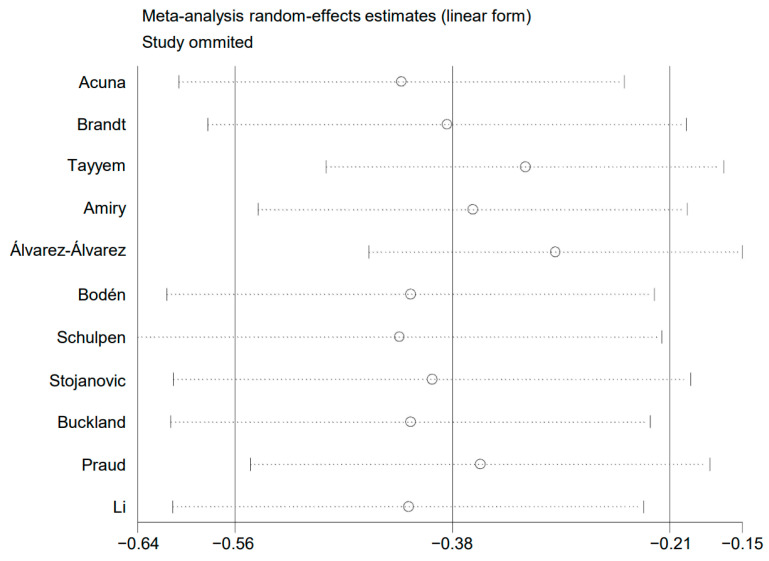
Plot of sensitivity analysis.

A sensitivity analysis was conducted. The pooled risk estimates were recalculated after removing a single study each time. No drastic change was found in the pooled risk estimates, as shown in Figure 6. This analysis confirmed that the results of the present meta-analysis were stable.

### 3.6. Publication Bias

**Figure 7:**
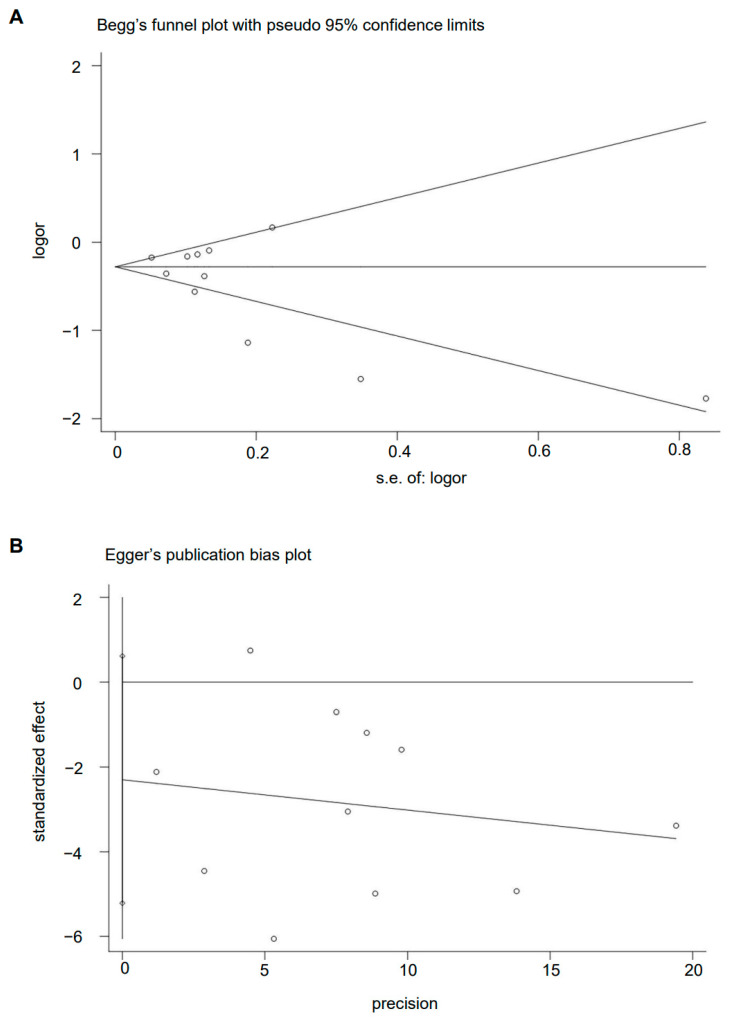
Plot of (**A**) Begg’s test and (**B**) Egger’s test.

The Begg’s funnel plot of MD pattern and GC risk showed basically symmetry (*p* = 0.436) (Figure 7A). There is also no evidence of the presence of publication bias among the studies according to the result of Egger’s linear regression test (*p* = 0.108) (Figure 7B).

## 4. Discussion

As far as we know, diet plays a key role in GC development, which is gradually attracting the attention of both oncologists and nutritionists in the field of cancer. Numerous meta-analyses have investigated the correlation between a specific food and GC risk [30,31]. Generally, a diet with highly salted food and red or processed meat increases the risk of GC, while a diet with fresh fruits, vegetables, and nuts may prevent GC [32,33,34,35].

In addition to particular items in the diet, GC risk is influenced by dietary patterns. The MD pattern is characterized by simple cooking and light flavors, with more emphasis on whole grains, vegetables, seafood, and fruits, and less emphasis on red meat and sweets, as recommended by nutritionists. Schwingshackl et al. [36] reported that the highest MD adherence led to a considerable decrease in the overall cancer mortality/incidence; however, there was no discernible change in GC risk. A case–control study from 2003 to 2015 in Italy found that the MD pattern and the individual components of the MD had a protective role against GC [7]. Subsequently, an updated meta-analysis that included this Italian study found an inverse correlation between the MD and GC risk [37]. Thus, the correlation between the MD and GC risk remains controversial and may be related to other influencing factors. Our meta-analysis first pooled several studies that mentioned the MD and its risk for GC. We found that adherence to the MD was significantly correlated with a lower GC incidence. It is speculated that inflammation-related responses may be involved in the possible biological mechanism of the MD’s protection against GC. The MD is rich in vitamins C and E, polyphenols, and folate, which promote its anti-inflammatory and antioxidant effects and thus inhibit multiple cancer-related biological pathways [38,39].

In this meta-analysis, the inverse association between MD and GC risk can be found in observational studies, including case–control and cohort studies. However, a recent review on vitamin D and cancer stated that observational studies using 25-hydroxyvitamin D concentrations drawn prior to cancer diagnosis are believed to be more precise than blood drawn closer to the time of cancer diagnosis [16]. In other words, case–control studies are more potent than cohort studies because long follow-up time reduces the effect estimates. Moreover, another study showed that case–control studies were considered the best type of study among observational studies due to the possibility of reverse causality [40]. A guideline on the analysis of nutrient effects also states that nutrient effects depend not only on the intake of the nutrient itself, but also on the duration of the follow-up period [41]. Although these conclusions are not from the diet, it is really inspiring to us. We performed the linear regression analysis of OR against follow-up time, showing that OR in our meta-analysis included both case–control and cohort studies and was indeed highly influenced by follow-up time (Figure 3). Therefore, the results of the meta-analysis of case–control studies served as the primary outcome of this study.

To the best of our knowledge, Helicobacter pylori infection is a major risk factor for GNCA; however, no positive correlation between H. pylori infection and GCA has been reported [42,43]. A previous study demonstrated that GCA and GNCA might have different risk factors [44]. Similarly, it remains unclear whether the relationship between the MD and GCA risk is different from that between the MD and GNCA risk. A cohort study conducted in the Netherlands with 120,852 participants reported that a higher MD adherence had an association with a lower risk of both GCA and GNCA [27]. Another study observed an inverse correlation between the MD and GC subtypes [8]. Our meta-analysis assessed the effect of the MD on GC subtypes. MD adherence was correlated with a lower risk of GCA and GNCA. However, as we mainly investigated the relationship between MD and GC subtypes in case–control studies, and the number of case–control studies in this study was small, heterogeneity was inevitable. According to GLOBOCAN 2020 estimates, the incidence of GC is twice higher in males than in females [1]. Previous studies have shown that a higher MD adherence was linked to a remarkable reduction in the prevalence of GC subtypes in men, in contrast to the nonsignificant inverse association observed in women [9,27]. The present study therefore investigated the influence of MD adherence on GC in men and women and found that our findings are consistent with theirs (Figure 5B). However, due to literature data limitations, the subgroup analysis of MD and GC risk by gender was conducted only in two countries along the Mediterranean coast with a predominantly MD, that is, Spain and Italy [7,29]. As for subgroup analysis by geographic location, although the protective effect of MD against GC was more significantly in Asia, Asian countries included only two studies with a total of 756 participants in this meta-analysis [24,25] (Figure 5A). But a similar protective role was not found in the United States (Figure 5C). Cancer burden, genetics, and environmental factors vary in different countries, so an uneven geographic distribution may lead to heterogeneity. Therefore, more studies are needed to verify these findings.

Participants’ diet data were collected from the included studies using a food frequency questionnaire [45]. The MD score (MDS) was used to evaluate the level of adherence to the MD, and a high MDS indicated high MD adherence. However, various dietary scores were used in the included studies. Three studies adopted a relative MDS and an alternate MDS [8,27,28]. Few studies have used a modified version of the traditional scoring of the MD [9,29]. A significant difference between these scoring systems was their cut-off value for defining moderate alcohol consumption. Another difference was related to healthy fat intake. More relevant details are described in the previous literature [46,47,48,49]. Castelló et al. [50] calculated MD’s eight main components via principal component analysis without a rotation of the variance–covariance matrix [51,52]. In an Italian study [7], the degree of MD adherence was evaluated using the literature-based MD adherence score developed by Sofi et al. [53]. On the basis of the abovementioned reports, the diversity of dietary scores for the MD pattern is an obvious limitation of our meta-analysis.

Other limitations also affected the interpretation of the results. First, the adjustment factors in the included studies were inconsistent, leading to a large difference in the value of OR and HR. This affected our statistical results. Second, all eligible studies were prospective cohort or case–control studies without explicit intervention, and the number of included studies was too small. Third, MD may differ in each country, which also resulted in high heterogeneity. Studies included in this meta-analysis used different MD scales and diet pattern analyses, causing it to not be possible to compare results and draw a causality among different scales. However, our results showed a high relationship between adherence to different types of MD scales and GC risk. We hope that future studies would minimize these biases and verify our results. Moreover, additional randomized controlled studies with larger sample sizes and better design are needed to investigate causality.

## 5. Conclusions

The present meta-analysis demonstrates that high adherence to MD is correlated with a lower GC risk. Additionally, higher MD adherence is associated with a decreased risk of both GCA and GNCA. Future well-designed studies are required to confirm our results.

## Figures and Tables

**Table 1:** Characteristics of the included studies.

First Author [Ref], Year	Region	Design	Population; Follow-Up or Cases/Controls	Age (Years)	Gender	Components of Score	Adjustment	Multivariate-Adjusted OR/HR (95% CI)	NOS
Acuna [22], 2023	United states	Prospective cohort	176,752; 19.2 y	45–75	M/F	1. ↑ vegetables (including potatoes); 2. ↑ fruits; 3. ↑ nuts; 4. ↑ legumes; 5. ↑ fish; 6. ↑ whole grains; 7. ↑ MUFA/SFA ratio; 8. ↔ alcohol; 9. ↓ red and processed meat	Age, sex, race, smoking status, pack years of cigarette smoking, BMI, total energy intake	GCA: HR: 1.56 (0.95, 2.54); GNCA: HR: 0.99 (0.78, 1.26)	8
Brandt [23], 2022	The Netherlands	Prospective cohort	120,852; 20.3 y	55–69	M/F	1. ↑ vegetables (without potatoes); 2. ↑ legumes; 3. ↑ fruits; 4. ↑ nuts; 5. ↑ whole grains; 6. ↑ fish; 7. ↑ MUFA/SFA ratio; 8. ↓ red and processed meat	Age, sex, smoking frequency and duration, highest level of education, family history, chronic diseases, energy intake, BMI, physical activity, alcohol intake	GCA: HR: 0.57 (0.34, 0.96) for highest score category (6–8) versus lowest score category (0–3); GNCA: HR: 0.72 (0.55, 0.96) for highest score category (6–8) versus lowest score category (0–3)	9
Buckland [28], 2015	Europe	Prospective cohort	461,550; 11.4 y	25–70	M/F	1. ↑ vegetables; 2. ↑ legumes; 3. ↑ fruit (including nuts and seeds); 4. ↑ cereals; 5. ↑ fish and seafood; 6. ↑ olive oil; 7. ↓ meat; 8. ↓ dairy products	Total energy intake, education level, BMI, physical activity level	HR: 0.87 (0.69, 1.09) for highest score category (≥8) versus lowest score category (<8)	7
Bodén [9], 2019	Sweden	Prospective cohort	100,881; 15.0 y	30–60	M/F	1. ↑ vegetables and potatoes; 2. ↑ fruit and juices; 3. ↑ whole-grain cereals; 4. ↑ fish and fish products; 5. ↑ MUFA+PUFA/SFA ratio; 6. ↔ alcohol intake; 7. ↓ meat and meat products; 8. ↓ dairy products	Energy intake, BMI, physical activity, smoking, educational status	HR: 0.85 (0.69, 1.03) per one tertile increase	6
Li [8], 2013	United States	Prospective cohort	49,468; 11.0 y	51–70	M/F	1. ↑ vegetables; 2. ↑ legumes; 3. ↑ fruit; 4. ↑ nuts; 5. ↑ whole grains; 6. ↑ fish; 7. ↑ MUFA/SFA ratio; 8. ↓ red and processed meat; 9. ↔ alcohol	Age, sex, race, smoking, education, BMI, vigorous physical activity, usual activity, total energy intake	GCA: HR: 1.10 (0.76, 1.61) for fifth versus first quintile; GNCA: HR: 0.75 (0.52, 1.09) for fifth versus first quintile	8
Schulpen [27], 2019	The Netherlands	Case–cohort	120,852; 20.3 y	55–69	M/F	1. ↑ vegetables; 2. ↑ fruits; 3. ↑ nuts; 4. ↑ legumes; 5. ↑ fish; 6. ↑ whole grains; 7. ↑ MUFA/SFA ratio; 8. ↓ red and processed meat	Age at baseline, sex, cigarette smoking status, cigarette smoking frequency, cigarette smoking duration, BMI, total daily energy intake, alcohol consumption, highest level of education, non-occupational physical activity, and family history of gastric cancer	GCA: HR: 0.86 (0.71, 1.04); GNCA: HR: 0.83 (0.73, 0.93)	7
Tayyem [24], 2022	Jordan	Case–control	172/314	Case: 54.1 ± 1.0; Control: 54.0 ± 0.7	M/F	1. ↑ fruits and juices; 2. ↑ vegetables; 3. ↑ lentils; 4. ↑ dairy products; 5. ↑ olive oil; 6. ↓ meat and meat products; 7. ↓ drinks and snacks	Age, gender, BMI, smoking, marital status, total energy intake, education level, family history, physical activity	OR: 0.212 (0.107, 0.419) for forth versus first quartile	6
Amiry [25], 2022	Afghanistan	Case–control	90/180	20–75	M/F	1. ↑ vegetables; 2. ↑ fruits; 3. ↑ nuts; 4. ↑ legumes; 5. ↑ fish; 6. ↑ whole grains; 7. ↑ MUFA/SFA ratio; 8. ↓ red and processed meat; 9. ↓ dairy products	Age, sex, physical activity, marriage status, smoking usage, toothbrushing, job, education, alcohol usage, BMI	OR: 0.17 (0.03, 0.80) for highest tertile versus lowest tertile	7
Álvarez-Álvarez [26], 2021	Spain	Case–control	354/3040	20–85	M/F	1. ↑ fruits; 2. ↑ vegetables (leafy, fruiting root, other); 3. ↑ legumes; 4. ↑ boiled potatoes; 5. ↑ fish (white and oily); 6. ↑ seafood/shellfish; 7. ↑ olives and vegetable oil; 8. ↓ juices	Sex, age, education, family history of gastric cancer, tobacco status, total energy consumed, BMI, NSAIDs intake, physical activity	OR: 0.32 (0.22, 0.46) for highest tertile versus lowest tertile	8
Stojanovic [7], 2017	Italy	Case–control	223/223	NA	M/F	1. ↑ fruit; 2. ↑ vegetables; 3. ↑ legumes; 4. ↑ fish; 5. ↓ meat and meat products; 6. ↔ alcohol	Sex, tobacco smoking, total energy intake	OR: 0.70 (0.61, 0.81)	6
Praud [29], 2013	Italy	Case–control	999/2628	19–80	M/F	1. ↑ cereals; 2. ↑ fruit; 3. ↑ vegetables; 4. ↑ legumes; 5. ↑ fish; 6. ↑ MUFA/SFA ratio; 7. ↓ milk (including dairy products); 8. ↓ meat (including meat products); 9. ↔ alcohol	Age, sex, study, year of interview, education, BMI, tobacco smoking, family history, total energy intake	OR: 0.57 (0.45, 0.70) for score ≥ 6 versus score ≤ 3	7

Abbreviations: MUFA, monounsaturated fat; PUFA, polyunsaturated fat; SFA: saturated fat; BMI, body mass index; M, male; F, female; NA, not available; GCA, gastric cardia adenocarcinoma; GNCA, gastric non-cardia adenocarcinoma; OR, odd ratio; HR, hazard ratio; CI, confidence interval; NOS, Newcastle–Ottawa Scale.

**Table 2:** Subgroup analysis for the source of heterogeneity.

Subgroups	No. of Studies	OR (95% CI)	Heterogeneity Test	
			I^2^	*p* Value
**Geographic location**				
Asia	2	0.21 (0.11, 0.39)	0.0%	0.81
Europe	3	0.52 (0.36, 0.76)	87.3%	<0.001
**Gender**				
Male	2	0.78 (0.65, 0.93)	75.5%	0.04
Female	2	0.83 (0.68, 1.01)	57.1%	0.13

## Data Availability

Not applicable.

## Supplementary Materials

The following supporting information can be downloaded at: https://www.mdpi.com/article/10.3390/nu15173826/s1, Table S1: Search strategy.
