# Supplementary material for: Adherence to the Mediterranean Diet and Risk of Gastric Cancer: A Systematic Review and Meta-Analysis
Source: Nutrients. 2023 Sep 1;15(17):3826. doi: 10.3390/nu15173826 (PMC10489619; doi:10.3390/nu15173826)
Supplement: Supplementary file 1 [file nutrients-15-03826-s001.zip › Supplementary Materials.pdf]

nutrients

MDPI

Supplementary Materials: Adherence to the Mediterranean Diet and Risk of Gastric Cancer: A Systematic Review and Meta-Analysis

Xiao Bai, Xue Li, Siqi Ding and Dongqiu Dai

Table S1. Search strategy

Search	Query	Items
**PubMed**		
#1	(Diet, Mediterranean[MeSH Terms]) OR (Diets, Mediterranean) OR (Mediterranean Diet) OR (Mediterranean Diet Score) OR (Dietary Pattern)	213007
#2	(Stomach Neoplasms[MeSH Terms]) OR (Stomach Neoplasm*) OR (Gastric Neoplasm*) OR (Stomach Cancer*) OR (Gastric Cancer*) OR (Gastric Adenocarcinoma)	177485
#3	#1 AND #2	**1139**
**Embase**		
#1	'mediterranean diet'/exp OR 'dietary pattern'/exp OR ' mediterranean diet score'	17492
#2	'stomach cancer'/exp OR 'stomach adenocarcinoma'/exp OR 'gastric cancer' OR 'gastric neoplasm'	178206
#3	#1 AND #2	**98**
**Cochrane Library**		
#1	MeSH descriptor: [Diet, Mediterranean] explode all trees	756
#2	("Mediterranean Diet") OR ("Mediterranean Diet Score") OR ("Dietary Pattern")	4629
#3	#1 OR #2	4629
#4	MeSH descriptor: [Stomach Neoplasms] explode all trees	3449
#5	("Stomach Neoplasm*") OR ("Gastric Neoplasm*") OR ("Stomach Cancer*") OR ("Gastric Cancer*") OR ("Gastric Adenocarcinoma")	11943
#6	#4 OR #5	11943
#7	#3 AND #6	**37**
**Web of Science**		
#1	TS=("Diet, Mediterranean" OR "Mediterranean Diet" OR "Mediterranean Diet Score" OR "Dietary Pattern")	99764
#2	TS=("Stomach Neoplasms" OR "Stomach Neoplasm" OR "Gastric Neoplasm*" OR "Stomach Cancer*" OR "Gastric Cancer*" OR "Gastric Adenocarcinoma")	308307
#3	#1 AND #2	**713**
